# The Impact of Hyperuricemia on Blood Pressure Elevation in Young Adults: A Systematic Review and Meta-Analysis

**DOI:** 10.21315/mjms-03-2026-167

**Published:** 2026-06-30

**Authors:** Enrico Yusuf, Ahmad Mahdi Rezkiansyah, Amanda Putri Gunarso, Ruben Almando Panjaitan

**Affiliations:** Faculty of Medicine, Universitas Pembangunan Nasional “Veteran” Jakarta, South Jakarta, DKI Jakarta 12450, Indonesia

**Keywords:** hyperuricemia, hypertension, young adults, uric acid, meta-analysis

## Abstract

The prevalence of hypertension among young adults is increasing globally. Although hyperuricemia is a widely recognised cardiovascular risk factor in older populations, its specific association with elevated blood pressure in young adults (< 40 years) remains under-investigated. Following PRISMA 2020 guidelines, the protocol was registered on the Open Science Framework. A comprehensive search was conducted across seven databases to identify observational studies involving adults aged 18 to 40 years. Methodological quality was assessed using the Joanna Briggs Institute Critical Appraisal Checklist. Random-effects meta-analyses were performed to estimate pooled mean differences (MDs) in systolic blood pressure (SBP) and diastolic blood pressure (DBP). Of the 1,165 identified records, 12 studies met the qualitative inclusion criteria. Nine studies were later excluded from the quantitative analysis because of incompatible data reporting, mismatched outcomes, or reversed predictor-outcome models. Thus, three high-quality studies (*N* = 4,733) with low risk of bias were included. The pooled analysis demonstrated that young adults with hyperuricemia had significantly higher blood pressure compared to normouricemic controls. The pooled MD was 12.06 mmHg (95% CI: 1.76, 22.35; *P* = 0.02) for SBP and 6.44 mmHg (95% CI: 2.83, 10.05; *P* = 0.0005) for DBP, with significant heterogeneity (*I*^2^ > 95%). Meta-analytic evidence confirms that hyperuricemia is significantly associated with elevated SBP and DBP in young adults. These findings support the use of serum uric acid as an early biomarker for assessing hypertension risk. Implementing early screening in young populations is advised to enhance cardiovascular prevention efforts.

## Introduction

Hypertension is a major health concern for older populations (1). Over the past decade, clinicians have observed a steady and alarming rise in the prevalence of hypertension among young adults (2). Early-onset hypertension is particularly concerning because prolonged exposure to elevated blood pressure may accelerate vascular damage and increase the lifetime risk of cardiovascular disease, stroke, chronic kidney disease, and other adverse health outcomes (3). The development of hypertension in young adults is influenced by obesity, physical inactivity, and high sodium intake, which explains many of these cases, they fail to account for the entire pathophysiological picture. This diagnostic gap suggests that other, less obvious metabolic risk factors might play a major role in younger patients (4).

Among these potential determinants, serum uric acid (SUA) has received increasing attention in recent years (5). Clinically, hyperuricemia is mainly known for its traditional association with gout and kidney stones (5). Hyperuricemia has traditionally been connected to gout, nephrolithiasis, and metabolic disorders; however, it is also associated with increased risks of adverse cardiovascular events in older patients with multiple comorbidities (3). Yet the specific pathophysiological role in young, generally healthy adults remains the subject of active debate. Experimental models show that high levels of circulating uric acid can directly damage endothelial cells, induce systemic oxidative stress, and irregularly activate the renin-angiotensin-aldosterone system (RAAS) (5, 6). These extensively documented biological pathways strongly indicate that high uric acid levels are not just indicators but also active causes that may directly contribute to increased blood pressure (7).

Despite these biological plausibilities, current evidence from human observational studies on SUA remains very mixed and inconclusive. Some researchers argue that elevated SUA serves as a strong, independent predictor of early-stage hypertension, potentially preceding clinical blood pressure changes by several years. Conversely, others believe it is merely an innocent bystander or simply a byproduct of broader metabolic syndrome and insulin resistance (5). The confounding effects of ageing and long-standing chronic diseases in older cohorts make it difficult to isolate the true cardiovascular impact of uric acid. Therefore, investigating this relationship specifically in young adults offers a unique opportunity to evaluate the independent vascular effects of hyperuricemia before other age-related comorbidities develop.

Because of this ongoing uncertainty in the literature, establishing a clearer clinical consensus is urgently needed to guide early cardiovascular screening and targeted preventative strategies. A comprehensive synthesis of the available data is essential to determine whether routine uric acid monitoring can improve risk stratification in younger demographic groups. Therefore, the primary objective of this systematic review and meta-analysis is to evaluate the existing observational evidence and quantify the strength of the association between hyperuricemia and elevated blood pressure among young adults (aged 18 to 40 years). Collectively, these processes may contribute to vascular dysfunction and increased peripheral resistance, thereby potentially promoting the development of hypertension.

## Methods

### Study Design and Registration

The systematic review and meta-analysis were conducted in accordance with the Preferred Reporting Items for Systematic Reviews and Meta-Analyses (PRISMA) 2020 guidelines (8). To ensure methodological transparency and mitigate reporting bias, the full review protocol was prospectively registered with the Open Science Framework (OSF) registry (DOI: 10.17605/OSF.IO/2BTQ4).

### Literature Search

A comprehensive and systematic literature search was performed across seven major electronic databases: PubMed, Scopus, Cochrane Library, EBSCOhost, OpenAlex, ProQuest, and ScienceDirect. The electronic search encompassed all articles published from the inception of each database up to 11 March 2026, and was independently conducted by two investigators (EY and AM). The selected databases were chosen to maximise the identification of relevant observational studies from both medical and multidisciplinary sources.

### Search Strategy

The search strategy was developed around three key concepts: i) exposure (“hyperuricemia” OR “uric acid” OR “serum urate”); ii) outcome (“hypertension” OR “blood pressure”); and iii) population (“young adult” OR “early onset” OR “students”). This primary search strategy was appropriately adapted to accommodate the specific syntax, wildcards (*), and indexing vocabularies (such as MeSH terms and TITLE-ABS-KEY field codes) required by each of the seven respective databases. The detailed search strings utilised for all databases are presented in Appendix 1.

### Eligibility Criteria

Before screening the literature, specific inclusion and exclusion criteria were set. Eligible studies had to be observational (such as cross-sectional, cohort, or case-control), focusing on young adult populations, primarily defined as individuals under 40 years of age. Furthermore, included studies had to evaluate the association between SUA levels and blood pressure, and provide sufficient quantitative data to compare a hyperuricemic group with the normouricemic control group. Conversely, animal experiments, in vitro studies, case reports, review articles, and research exclusively involving paediatric or elderly patients were excluded.

### Data Extraction and Quality Assessment

Data were independently extracted by two reviewing authors (AP and RA). Extracted information included author names, publication year, sample size, and blood pressure outcomes. Data regarding multivariate risk estimates (such as adjusted odds ratios, hazard ratios, and risk ratios) comparing the highest versus lowest uric acid quartiles were extracted for qualitative synthesis. Methodological quality and risk of bias were meticulously assessed using the Joanna Briggs Institute (JBI) Critical Appraisal Checklist for observational studies (9). Any discrepancies arising during screening, data extraction, or quality assessment processes were resolved through consultation with the lead investigator (EY) or through team discussions involving all authors (EY, AM, AP, and RA) until full agreement was achieved.

### Statistical Analysis

Statistical analyses and quantitative data syntheses were performed using Review Manager (RevMan) software (10). To maintain consistency across studies, standardised comparisons across the systematically reviewed literature, SUA levels reported in the International System of Units (μmol/L) were uniformly converted to conventional units (mg/dL) using a standard conversion factor (1 mg/dL = 59.48 μmol/L) (11).

For the quantitative meta-analysis, the focus was on continuous blood pressure parameters—systolic and diastolic blood pressure—which are universally measured in millimetres of mercury (mmHg). Pooled effect sizes for continuous outcomes were calculated using the weighted mean difference (MD) alongside 95% confidence intervals (CIs). The MD was strictly selected because all included quantitative studies assessed the blood pressure outcomes on the exact same scale (12).

Data pooling was conducted utilising the inverse-variance random-effects model. This statistical technique weights individual studies based on their sample size and variance. The random-effects approach was considered fundamentally appropriate over a fixed-effects model because of the inherent clinical and methodological differences in the source populations, including varying definitions of the “young adult” age group (from 18 to 45 years), different baseline demographics, and diverse observational study designs.

Furthermore, statistical heterogeneity among the included studies was formally assessed using the *I*^2^ statistic. The *I*^2^ value quantifies the percentage of total variability across the pooled studies attributable to true between-study differences rather than mere sampling chance. In our analysis, an *I*^2^ value exceeding 50% was interpreted as indicating substantial heterogeneity (13). Finally, an alpha level of < 0.05 and a *P*-value < 0.05 were considered statistically significant for all quantitative measures.

## Results

The initial search across seven databases yielded 1,165 potentially relevant records. After removing duplicates and screening titles and abstracts, 12 observational studies met the established strict criteria and were included in the qualitative systematic review. The baseline characteristics of the 12 included studies are summarised in Table 1, and their key findings on the association between SUA and hypertension are presented in Table 2. However, due to missing statistical parameters, only three studies provided sufficient comparable data for the quantitative meta-analysis, comprising a total pooled sample of 4,733 young adult subjects. All three included studies showed a low risk of bias based on the JBI Critical Appraisal. The detailed study selection process is illustrated in the PRISMA flow diagram (Figure 1).

The 12 studies included in the present systematic review represent a diverse global demographic, encompassing populations from Asia (China, Taiwan, Malaysia, Pakistan), the Americas (USA, Mexico), and Europe (Italy, Germany). The study designs varied widely, including prospective and retrospective cohort studies, cross-sectional surveys, and case-control studies, with data collected from military personnel, university students, and community-based volunteers.

The vast majority of synthesised evidence strongly supports a positive association between elevated SUA and elevated blood pressure in young adults. Crucially, large-scale prospective cohort studies provide robust temporal evidence. For instance, Thomas et al. (14) (*N* = 3,890) and Gaffo et al. (15) (*N* = 4,682) demonstrated that baseline SUA levels independently predicted the future incidence of hypertension, with Gaffo reporting a hazard ratio (HR) of 1.25 for young men. Similar findings were reported by Chen et al. (16), who observed an association between elevated SUA levels and an increased risk of hypertension in a cohort of 22,466 military personnel, particularly among men (HR: 1.65).

Cross-sectional and case-control data closely reflected the longitudinal results. Research on hypertension risk showed notably high odds ratios (OR). For example, Kammar-García (17) observed a massive risk increase (OR: 6.80; 95% CI: 1.10, 46.0) among university students, while Zhang et al. (18) reported an OR of 4.06 (95% CI: 2.11, 7.81) in a hospital-based young cohort. Similarly, large community and military cross-sectional surveys by Lin et al. (19) (*N* = 7,504), Wang et al. (20) (*N* = 1,869), and Krupp et al. (21) (*N* = 3,050) consistently demonstrated increased hypertension odds ranging from 1.57 to 1.71. Furthermore, studies by Sidoti et al. (22) and Umar et al. (23) observed that SUA is a continuous determinant of blood pressure, meaning that even subclinical increases in uric acid levels correlate directly with increases in both systolic and diastolic readings.

Despite the overwhelming consensus, one study presented contrasting results. Mustapha et al. (24) found no significant independent association (OR: 1.00) between hyperuricemia and hypertension in a Malaysian primary care cohort. This isolated discrepancy may be attributed to specific cohort characteristics, sample size limitations (*N* = 132), or differences in methodologies for adjusting for metabolic confounders. Overall, however, the qualitative synthesis consistently identifies SUA as a key driver of early-onset vascular changes.

In the quantitative meta-analysis, young adults with hyperuricemia had higher systolic blood pressure (SBP) than normouricemic controls, with a pooled MD of 12.06 mmHg (95% CI: 1.76, 22.35; *P* = 0.02). Substantial heterogeneity was observed across studies (*I*^2^ = 99%) (Table 3). Similarly, hyperuricemia was associated with higher diastolic blood pressure (DBP), showing a pooled MD of 6.44 mmHg (95% CI: 2.83, 10.05; *P* = 0.0005), also accompanied by high heterogeneity (*I*^2^ = 96%) (Table 4).

## Discussion

This meta-analysis clearly demonstrates that elevated SUA is not merely a benign metabolic byproduct or a simple risk factor for gout in older adults (3). It also plays a crucial, active role in increasing both systolic and DBP elevations in young, otherwise healthy individuals. By pooling data from over 4,700 young adults and synthesising qualitative evidence from diverse global cohorts, a stark and consistent contrast was identified: individuals with early-onset hyperuricemia exhibit substantially higher blood pressure parameters than their normouricemic peers. This finding fundamentally challenges the outdated clinical habit of ignoring asymptomatic high uric acid in young patients (26).

The biological mechanisms explaining how uric acid raises blood pressure are compelling and well documented in experimental models (6). Current evidence points to a “two-phase” mechanism of uric acid-induced hypertension (7). Initially, excess uric acid enters endothelial cells, creating a hostile environment by inducing oxidative stress and significantly decreasing endothelial nitric oxide levels (5). This lack of nitric oxide prevents vasodilation, causing the blood vessels to stiffen. Simultaneously, hyperuricemia directly stimulates the RAAS and promotes the proliferation of vascular smooth muscle cells (5). This dual hit—endothelial dysfunction combined with RAAS overactivity—creates the perfect storm for early-onset hypertension. If left untreated, this initial reversible phase eventually transitions into a late phase characterised by irreversible renal microvascular disease and salt-sensitive hypertension, where lowering uric acid no longer normalises blood pressure (27).

The magnitude of blood pressure elevation observed in the quantitative synthesis—a pooled MD of 12.06 mmHg for systolic and 6.44 mmHg for diastolic blood pressure—supports the severe risk estimates reported in individual cohorts. These findings strongly align with and build upon previous landmark research, most notably the comprehensive meta-analysis by Grayson et al. (7). While Grayson and colleagues investigated incident hypertension across a broad age spectrum, their subgroup analysis revealed that the relative risk of developing hypertension due to hyperuricemia was significantly higher in younger populations. The study reinforces this phenomenon by quantifying the precise hemodynamic burden placed on the youthful cardiovascular system.

Putting this severity into perspective, a 12 mmHg increase in systolic pressure in a young adult is not a trivial fluctuation. According to established cardiovascular risk paradigms derived from the Framingham Heart Study (28), a sustained increase of this magnitude prematurely ages the vascular system and exponentially drives up the lifetime risk of stroke and ischaemic heart disease. These pooled quantitative findings provide concrete, measurable hemodynamic evidence that validates the strikingly high OR (ranging from 2.98 to 6.80) observed in the qualitative cross-sectional analyses by Kammar-García et al. (17) and Ji et al. (25). In older adults, blood pressure is strongly influenced by decades of vascular ageing and cumulative comorbidities (3). In contrast, the isolated endothelial toxicity of uric acid serves as a primary pathogenetic trigger in the compliant blood vessels of young adults. This substantial blood pressure gap explains the temporal trajectory observed in the CARDIA cohort by Gaffo et al. (15), illustrating exactly how early-onset hyperuricemia translates into incident hypertension years afterwards.

### Clinical Significance of This Review

The clinical implications of these findings are straightforward but critical, presenting a vital window of opportunity for early intervention. Currently, routine cardiovascular health screenings for young adults rarely prioritise uric acid testing, instead focusing on traditional markers such as lipids and glucose (4). Given the current findings, physicians should see asymptomatic hyperuricemia as a clear warning sign of potential future cardiovascular problems. Catching this early allows doctors to implement aggressive lifestyle interventions—such as dietary modifications, reduced fructose intake, and weight management—before the reversible early phase of uric acid-induced endothelial dysfunction progresses to chronic, irreversible vascular remodelling (5).

### Strengths and Limitations

A major strength of the present review is the comprehensive synthesis of qualitative and quantitative data from a diverse global demographic, encompassing a robust pooled sample of more than 4,700 young adults. This improves the accuracy of the quantitative estimates. However, several limitations must be acknowledged. The most prominent issue is the high statistical heterogeneity (*I*^2^ > 95%) observed across the included meta-analysis models. This variation likely stems from clinical and methodological differences among the primary studies, including varying definitions of the “young adult” age bracket (ranging from under 30 to under 40 years), diverse baseline diets across different countries, and inconsistencies in blood pressure measurement protocols (29). Moreover, since the data were synthesised observationally, definitive proof of causality cannot be assured.

### Future Directions

The inability to fully rule out unmeasured residual confounders, such as genetic predisposition or hidden dietary habits, highlights a gap in current literature. Future large-scale randomised controlled trials focused on reducing uric acid in young adults are essential to determine if lowering SUA through medication or lifestyle changes leads to significant blood pressure normalisation.

## Conclusion

This systematic review and meta-analysis critically evaluate the association between SUA levels and blood pressure elevations in young adult populations. To support this objective, the synthesised evidence strongly confirms a significant, parallel relationship between early-onset hyperuricemia and markedly elevated systolic and DBP. Therefore, SUA should be considered merely as a benign metabolic byproduct or a risk factor for gout among older adults. It is also an active instigator and an early indicator of cardiovascular risk in younger populations. For clinical practice, these findings highlight the urgent need to consider routine uric acid screening for young adults. Early detection of high uric acid and management through targeted lifestyle interventions could play a crucial role in preventing the onset of chronic hypertension and its severe long-term complications.

## Figures and Tables

**Figure 1:**
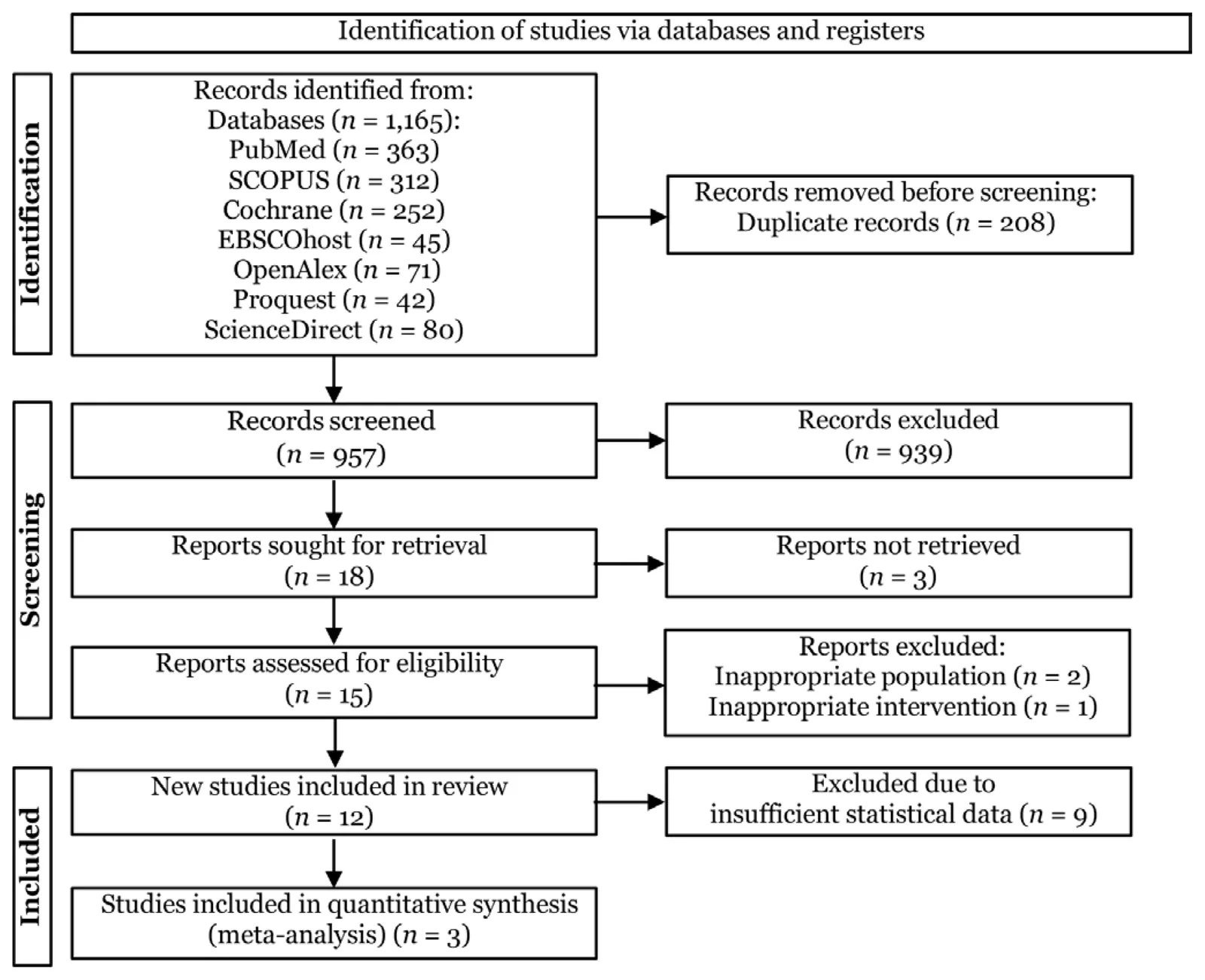
PRISMA flow diagram of the study selection process.

**Table 1:** Baseline characteristics of the 12 included observational studies.

Study	Country	Study design	Population setting	Sample size (*N*)	Mean age (years)^a^
Thomas et al. (14)	USA	Cohort (prospective)	Community cohort	3,890	24.7 ± 3.7
Gaffo et al. (15)	USA	Cohort (prospective)	Community cohort	4,682	24.8 ± 3.6
Chen et al. (16)	Taiwan	Cohort (retrospective)	Military personnel	22,466	< 40 (range)
Kammar-García et al. (17)	Mexico	Cross-sectional	University students	768	18.8 ± 1.4
Zhang et al. (18)	China	Case-control	Hospital-based	488	31.9 (cases)
Lin et al. (19)	Taiwan	Cross-sectional	Military cohort	7,504	28.9 ± 6.0
Wang et al. (20)	China	Cross-sectional	Community-based	1,869	43.0 (median)
Krupp et al. (21)	Germany	Cross-sectional	Population survey	3,050	34.8 (mean)
Sidoti et al. (22)	Italy	Cross-sectional	High school students	54	18 to 21 (range)
Umar et al. (23)	Pakistan	Cross-sectional	Outpatient volunteers	200	30.0 (median)
Mustapha et al. (24)	Malaysia	Case-control	Primary care clinic	132	34.0 (cases)
Ji et al. (25)	China	Cross-sectional	College students	285	20.4 ± 2.2

a Age is presented as mean ± standard deviation, median, or range, depending on the data reported by the primary study

**Table 2:** Key findings regarding the association between elevated SUA and hypertension outcomes.

Study	Key findings	Effect size (OR/HR) 95% CI
Thomas et al. (14)	SUA predicted HTN incidence by age 55	HR: 1.12 (blacks); 1.07 (whites)
Gaffo et al. (15)	Increased incident HTN risk	HR: 1.25 (men); 1.06 (women)
Chen et al. (16)	SUA predicted HTN in young men	HR: 1.17 (< 30 y); 1.65 (30 to 40 y)
Kammar-García et al. (17)	High SUA was a major predictor for elevated BP	OR: 6.80 95% CI: 1.10, 46.0
Zhang et al. (18)	Early-onset hyperuricemia increased HTN risk	OR: 4.06 95% CI: 2.11, 7.81
Lin et al. (19)	Elevated SUA significantly increased HTN risk	OR: 1.57 95% CI: 1.33, 1.86
Wang et al. (20)	Top quartile of SUA linked to pre-hypertension	OR: 1.71 95% CI: 1.21, 2.42
Krupp et al. (21)	Diet-independent relevance of SUA for high BP	OR: 1.71 95% CI: 1.10, 2.67
Sidoti et al. (22)	Uric acid acted as a significant continuous determinant of BP levels	NR
Umar et al. (23)	Positive correlation found between SUA and continuous BP variables	NR
Mustapha et al. (24)	No significant independent association found in this specific cohort	OR: 1.00
Ji et al. (25)	Hyperuricemia strongly associated with HTN	OR: 2.98 95% CI: 1.67, 5.29

SUA = serum uric acid; HTN = hypertension; BP = blood pressure; OR = odds ratio; HR = hazard ratio; CI = confidence interval; NR = not reported

**Table 3:** Pooled MD in SBP between young adults with hyperuricemia and normouricemia.

Study	Hyperuricemia					Normouricemia					Weight (%)	MD (95% CI)

	Mean		SD		*N*	Mean			SD	*N*		
Ji et al. (28)	152.4		11.2		101	120.5			11.9	184	24.8	31.90 (29.12, 34.68)
Kammar-García et al. (17)	121.5		12.7		189	113.9			13.3	579	25.0	7.60 (5.49, 9.71)
Lin et al. (19) - A	122.92		13.55		1720	118.17			13.21	1,583	25.2	4.75 (3.84, 5.66)
Lin et al. (19) - B	109.77		13.25		207	105.63			12.95	170	24.9	4.14 (1.49, 6.79)

Total (95% CI)				2,217						2,516	100.0	12.06 (1.76, 22.35)^a^

CI = confidence interval; *N* = sample size; SD = standard deviation; MD = mean difference.

a Overall effect size calculated using a random-effects model.

Heterogeneity: tau^2^ = 109.05; chi^2^ = 336.17, df = 3 (*P* < 0.00001); *I*^2^ = 99%. Test for overall effect: Z = 2.30 (*P* = 0.02).

**Table 4:** Pooled MD in DBP between young adults with hyperuricemia and normouricemia.

Study	Hyperuricemia			Normouricemia			Weight (%)	MD (95% CI)

	Mean	SD	*N*	Mean	SD	*N*		
Ji et al. (28)	83.9	10	101	69.5	8.4	184	23.9	14.40 (12.10, 16.70)
Kammar-García et al. (17)	74.7	7.5	189	69.8	9.8	579	25.5	4.90 (3.57, 6.23)
Lin et al. (19) - A	74.06	11.02	1,720	70.41	9.89	1,583	26.2	3.65 (2.94, 4.36)
Lin et al. (19) - B	67.21	10.31	207	63.95	9.3	170	24.5	3.26 (1.28, 5.24)

Total (95% CI)			2,217			2,516	100.0	6.44 (2.83, 10.05)^a^

a CI = confidence interval; *N* = sample size; SD = standard deviation; MD = mean difference.

Overall effect size calculated using a random-effects model. Heterogeneity: tau^2^ = 12.86; chi^2^ = 78.64, df = 3 (*P* < 0.00001); *I*^2^ = 96%. Test for overall effect: Z = 3.49 (*P* = 0.0005).

## Appendix

### Appendix 1 Detailed Search Strategies

Database: PubMed/Scopus/Cochrane Library/EBSCOhost/OpenAlex/ProQuest/ScienceDirect Date of search: Inception to 11 March 2026

**Table:**

Database	Search query	Hits
PubMed	(“Uric Acid”[Mesh] OR “Hyperuricemia”[Mesh] OR “serum uric acid” OR urate) AND (“Hypertension”[Mesh] OR “Blood Pressure”[Mesh] OR “high blood pressure”) AND (“Young Adult”[Mesh] OR “young adults” OR “early onset” OR “early adulthood” OR “college students” OR “university students”)	363
Scopus	TITLE-ABS-KEY ( ( “uric acid” OR “serum urate” ) AND ( hypertension OR “blood pressure” ) AND ( “young adult*” OR “early onset” ) AND ( cohort OR longitudinal OR prospective ) ) AND NOT ( preeclampsia OR pregnan* OR animal OR rat )	312
Cochrane Library	(“uric acid” OR “serum urate” OR hyperuric*) AND (hypertension OR “blood pressure”) AND (“young adult” OR “young adults” OR adolescent* OR “early onset” OR student*)	252
EBSCOhost	(TI “uric acid” OR AB “uric acid” OR TI “serum urate” OR AB “serum urate” OR TI hyperuric* OR AB hyperuric*) AND (TI hypertension OR AB hypertension OR TI “blood pressure” OR AB “blood pressure”) AND (TI “young adult*” OR AB “young adult*” OR TI “early onset” OR AB “early onset” OR TI student* OR AB student*) AND (TI cohort OR AB cohort OR TI longitudinal OR AB longitudinal OR TI prospective OR AB prospective)	45
OpenAlex	(“uric acid” OR “serum urate” OR hyperuricemia) AND (hypertension OR “blood pressure”) AND (“young adult” OR “early onset” OR “university student”) AND (cohort OR longitudinal OR prospective)	71
ProQuest	(TI(“uric acid” OR “serum urate” OR hyperuric*) OR AB(“uric acid” OR “serum urate” OR hyperuric*)) AND (TI(hypertension OR “blood pressure”) OR AB(hypertension OR “blood pressure”)) AND (TI(“young adult*” OR “early onset” OR student*) OR AB(“young adult*” OR “early onset” OR student*)) AND (cohort OR longitudinal OR prospective)	42
ScienceDirect	(“uric acid” OR “serum urate” OR hyperuricemia) AND (hypertension OR “blood pressure”) AND (“young adult” OR “early onset” OR students)	80
